# Cardiac Contractility Modulation Therapy in Patients with Amyloid Cardiomyopathy and Heart Failure, Case Report, Review of the Biophysics of CCM Function, and AMY-CCM Registry Presentation

**DOI:** 10.3390/jcm12031184

**Published:** 2023-02-02

**Authors:** Procolo Marchese, Francesca Gennaro, Giovanni Mazzotta, Claudia Acciarri, Stenio Amabili, Carlo Bonanni, Antonella D’Antonio, Domenico Delfino, Luca Di Vito, Manrico Partemi, Riccardo Pascucci, Andrea Romandini, Giancarla Scalone, Simona Silenzi, Pierfrancesco Grossi

**Affiliations:** Cardiology Unit, Mazzoni Civil Hospital, 63100 Ascoli Piceno, Italy

**Keywords:** cardiac contractility modulation, heart failure, amyloidosis

## Abstract

Cardiac amyloidosis may result in an aggressive form of heart failure (HF). Cardiac contractility modulation (CCM) has been shown to be a concrete therapeutic option in patients with symptomatic HF, but there is no evidence of its application in patients with cardiac amyloidosis. We present the case of TTR amyloidosis, where CCM therapy proved to be effective. The patient had a history of multiple HF hospitalizations due to an established diagnosis of wild type TTR-Amyloidosis with significant cardiac involvement. Since he was highly symptomatic, except during continuous dobutamine and diuretic infusion, it was opted to pursue CCM therapy device implantation. At follow up, a significant improvement in clinical status was reported with an increase of EF, functional status (6 min walk test improved from zero meters at baseline, to 270 m at 1 month and to 460 m at 12 months), and a reduction in pulmonary pressures. One year after device implantation, no other HF hospital admission was needed. CCM therapy may be effective in this difficult clinical setting. The AMY-CCM Registry, which has just begun, will evaluate the efficacy of CCM in patients with HF and diagnosed TTR amyloidosis to bring new evidence on its potential impact as a therapeutic option.

## 1. Background

Cardiac amyloidosis is primarily associated with aggregates of amyloidogenic proteins, which may be immunoglobulin light chain proteins (AL) or transthyretin proteins (TTR), in many cardiac structures causing different types of amyloidosis. It may result in an aggressive form of heart failure (HF) [1]. In addition to the studies currently ongoing, other therapeutic options that can work synergistically in this clinical setting should be tested. Randomized clinical trials showed that cardiac contractility modulation (CCM) therapy may be considered in patients with symptomatic HF despite optimal medical therapy (OMT), with Ejection Fraction (EF) between 25% and 45%, and without an indication for cardiac resynchronization therapy (CRT) [2,3,4]. We present the case of a patient with TTR amyloidosis, where CCM therapy proved to be effective. We reviewed the biophysics and molecular biology mechanisms underlying CCM function which led to the idea of designing a registry to further explore the efficacy of CCM in cardiac amyloidosis.

## 2. Case Report: Wild Type TTR

**Figure 1:**
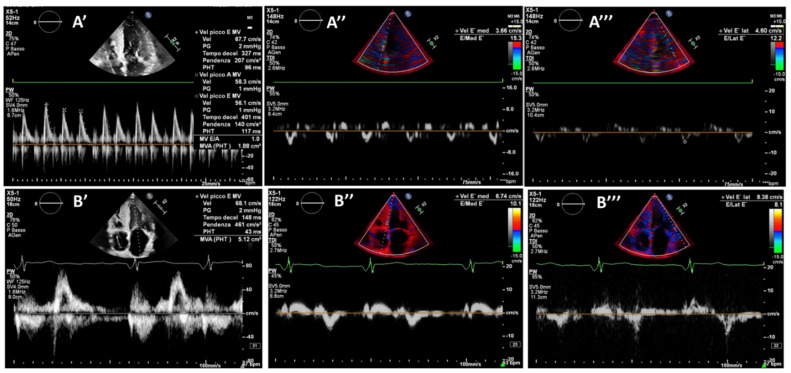
**Diastolic function improvement.** Baseline diastolic function E/A (**A′**), E/E′ septal (**A″**), E/E′ lateral (**A‴**); 12-month diastolic function E/A (**B′**), E/E′ septal (**B″**), E/E′ lateral (**B‴**).

The patient is a 67 year old male with a history of multiple HF hospitalizations due to an established diagnosis of wild type TTR-Amyloidosis for at least 6 years, with significant cardiac involvement requiring dobutamine and diuretic infusions. His baseline EF was 38%, with a restrictive diastolic pattern. After informed consent, the patient underwent CCM therapy device implantation in September 2020. Of note, two days after CCM implantation, the patient’s clinical status improved. Three months post-implant, the patient’s 6 min walk test (6MWT) was 320 m, and the EF was 43%. It was also possible to start therapy with beta-blockers and a low dose of sacubitril/valsartan, which were not tolerated before CCM. At the 6 month follow up, the EF was 48% and there was improvement in the echocardiographic diastolic pattern (pseudo-normal) and a reduction in pulmonary pressures (Figure 1).

At the 12 month follow up, for the 6MWT he walked 460 m. Furthermore, at that time point, the Kansas City Cardiomyopathy score improved from 11.1 at baseline to 82.8. At this point in time, the improvements allowed us to start Tafamidis. Echo and functional parameters have been stable, and no other HF hospital admission occurred during the 26 months of follow ups.

To the best of our knowledge, this is the first published case of cardiac amyloidosis successfully treated with CCM therapy. In other clinical settings it has been proven that the short- and long-term use of CCM therapy improves both the strength of the ventricular contraction and the pumping capacity of the heart by modulating myocardial contraction, thus improving the reported symptoms, and reducing HF hospitalizations [2,3,4]. 

## 3. Discussion and Review of CCM Function

In heart failure, mortality is still high despite the advances in medical and device therapy. Most patients receiving optimal medical therapy (OMT) have limited possibility possibly for up-titration and this is far more difficult in the clinical setting of cardiac amyloid because of intolerance to several HF drugs. 

ICD is not HF therapy and unfortunately only one-third of patients with HF have a QRS complex wider than 120 msec and would thus be suitable for CRT. Moreover, one-third of patients receiving CRT are non-responders, thus they remain symptomatic, despite OMT [5].

CCM therapy is a proper HF therapy and delivers high amplitude non-excitatory biphasic electrical signals during the myocardial refractory period. It is applicable for patients with NYHA class II or III status, an LVEF < 50% (per CE Mark), peak VO2 ≥ 10 mL/kg/min, and PVCs less than 10,000 per day. CCM is also suitable for patients with atrial fibrillation and non-responders to CRT.

The CCM implant procedure does not differ from pacemaker implantation, with the exception of the placement of two leads in the RV rather than one. It is performed using cephalic or subclavian vein access (often right sided because often there is already an ICD in place on the left side). Two active fixation leads are secured to the right ventricular septum at least 2–3 cm apart from each other and at least 3 cm from the defibrillation RV lead. The leads are used for sensing ventricular activity and for bipolar delivery of CCM signals. Electrical testing of the leads includes the standard testing for pacemaker leads with a higher focus on the sensing function. Active CCM treatment is generally programmed to be delivered daily for at least 7 h, in equally spaced one hour intervals throughout the day, targeting a minimum of 90% CCM therapy delivery [3,4,5,6,7,8].

## 4. CCM “Pharmacodynamic”: Are We Dealing with Quantum Medicine?

Prior studies have shown that when applied to isolated papillary muscles in vitro, CCM signals increase myocardial contractility [9]. The mechanism has been shown to fundamentally relate to an increase in action potential duration by CCM signals, which enhances trans-sarcolemmal calcium entry. This in turn causes calcium loading of the sarcoplasmic reticulum and increased calcium release to the myofilaments. Though the acute impact of CCM signals on contractile strength was shown to be limited to only the region of signal application, at three months, changes are documented to extend to regions remote from signal delivery. 

CCM action could be possibly due to production of a specific electromagnetic field (EMF) that modulates the quantum mechanics aspects of biological process of the HF [10]. 

Richard Friedman (1965 Nobel Prize in Physics “for their fundamental work in quantum electrodynamics, with deep-ploughing consequences for the physics of elementary particles”) used to say, “I think I can safely say that nobody understands quantum mechanics”. Based on this prestigious assumption, we felt emboldened in trying to theorize the biophysical background of CCM “pharmacodynamics”. 

In quantum theory, according to the Born-Oppenheimer Approximation assumption, EMFs interact more strongly with electrons because of their unusually high charge to mass ratio. Electrons are assumed to respond instantaneously compared to protons and heavier atomic nuclei because of their much smaller mass. In biological systems, therefore, it is reasonable to expect EMFs to interact initially with small subatomic particles and with whole molecules by specific electrical charged regions, leading to early-onset effects which are more likely due to enzymes function modulation and late-onset effects which are related with DNA interaction via specific electromagnetic response elements (EMREs). 

## 5. CCM Early-Onset Effects: Enzymes Modulation

Enzymes are large biological molecules, usually proteins, that speed up chemical reactions. Molecules that speed up chemical reactions, but are unchanged afterwards, are known as catalysts. The substances that enzymes act on with a remarkable specificity are known as substrates [11].

**Figure 2:**
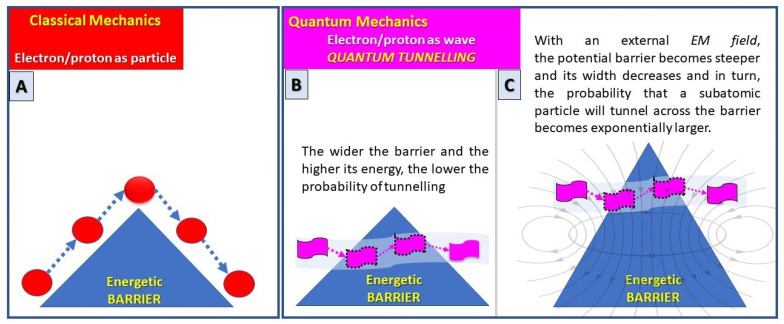
***Schematic representation of quantum tunnelling.*** (**A**) Old Transition state theory, aiming to understand how enzymes facilitate passage of the reaction over a static potential-energy barrier to proceed from reactants to products. (**B**) In quantum mechanics, particles don’t have defined positions in space, but their position is instead defined by a diffuse wave function. This is known as an aspect of Heisenberg’s uncertainty principle which implies the possibility that the edges of particle waves leak through classical barriers, a process known as quantum tunnelling. (**C**) Effect of external EMF could enhance the probability that a particle will tunnel across the barrier.

The focus of the last 50 years’ experiences is known as transition state theory (TST), aiming to understand how enzymes facilitate passage of the reaction over a static potential-energy barrier to proceed from reactants to products [12] (Figure 2A). However, recent studies have revealed that passage through, rather than over, the barrier can occur, and that quantum mechanical phenomena can play a crucial role in enzyme action [13,14,15]. Matter has particle-like properties but can also be considered as having wave-like properties (especially those with smaller mass): this is known as the wave-particle duality of matter. Specifically, according to quantum mechanics, particles do not have defined positions in space, but their position is instead defined by a diffuse wave function. This is known as an aspect of Heisenberg’s uncertainty principle, which implies the possibility that the edges of particle waves leak through classical barriers, a process known as quantum tunnelling.

Electrons can travel large distances (up to 3 nm) through proteins despite the latter being electrical insulators. This paradox can be explained in terms of the wave-like properties of the electron that allow it to pass via quantum tunnelling through regions from which it would be excluded by its particle-like nature [16]. 

Quantum tunnelling may also play an important role in driving enzyme-catalysed reactions, especially for the transfer of small nuclei, such as hydrogen. The pathway from reactants to products in an enzyme-catalysed reaction may not need to pass over the barrier, as in TST with particle-like behaviour, but could pass through the barrier [17] (Figure 2B). The wider the barrier and the higher its energy, the lower the probability of tunnelling. It has been also demonstrated that when an external EMF is applied, the potential barrier outside the conductor becomes steeper and its width decreases for an electron with a given kinetic energy. In turn, the probability that an electron will tunnel across the barrier becomes exponentially larger [14,15] (Figure 2C). 

**Figure 3:**
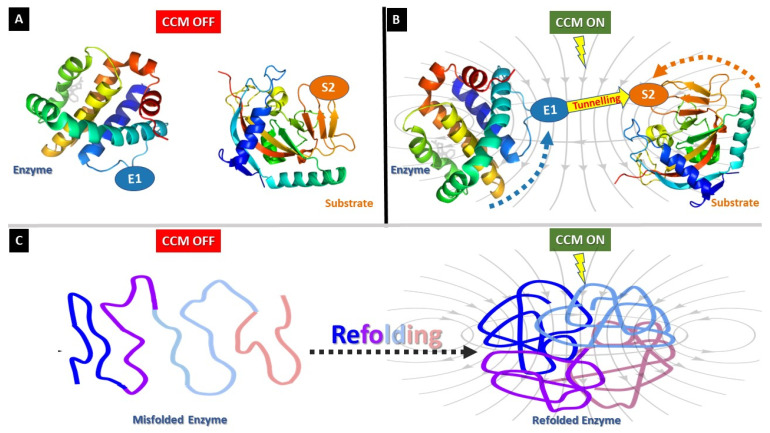
***Schematic representation of CCM induced EM field mechanism of action on enzymes.*** EMFs impact may also increase the probability of quantum tunnelling by inducing proper orientation of substrates and enzymes toward each other (**A**,**B**). In addition, EMF can induce refolding of denatured enzymes which can further enhance the activity (**C**).

Accordingly, low frequency electric and magnetic fields were shown to affect enzyme function. Notably, both fields accelerated the reaction only when the intrinsic chemical forces are relatively weak and when enzyme activity was low [10]. However, the exact mechanism of EMF action on enzymes at atomic level is not fully understood. It might be due to creation of additional active sites or positive modification of existing active sites/overall globular structure. EMF impact may also increase the probability of quantum tunnelling by inducing proper orientation of substrates and enzymes toward each other (Figure 3A). In addition, EMF can induce refolding of denatured enzymes, which can further enhance the activity [18,19,20,21] (Figure 3B).

Remarkably, this is an early-onset effect. Sun et al. demonstrated that EMFs could induce the phosphorylation of stress-activated protein kinase (SAPK) extracted from Chinese hamster lung cells within 15 min in a time- and intensity-dependent manner [22].

**Figure 4:**
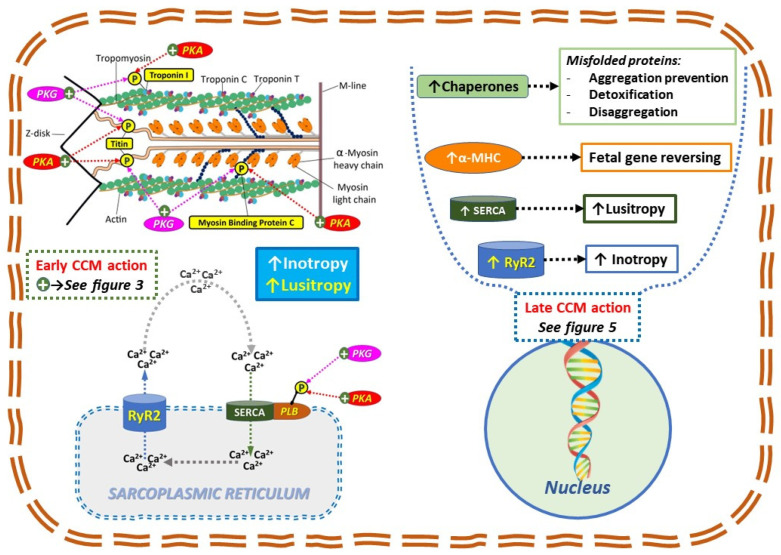
Schematic representation of early and late onset effects on CCM function (see explanation in the text. The left panel refers to early onset effect which are related to enzyme modulation by CCM induced EMF (green positive circle refers to mechanism depicted in Figure 3). An increase in the phosphorylation state of troponin and myosin binding protein C leads to positive inotropy. An increase in the phosphorylation state of PLB and titin leads to positive lusitropy. PKA = phosphokinase A; PKB = phosphokinase B. The right panel refers to late onset effects which are related to DNA transcription modulation by CCM induces EMF (see Figure 5 for detailed mechanism on DNA strands). There is a substantial fetal gene reverse remodelling by increasing the down regulated RyR2, SERCA, and α-MHC. Moreover, the increase in Chaperones transcription (such as HSP70) has several positive effects such as aggregation prevention, detoxification, and disaggregation of misfolded proteins.

Consistently, it has been proven that CCM EMF acts early on specific phosphorylation enzymes, such as the one that enhanced the phosphorylation state of phospholamban (PPL) [23] within just 2 h of signal application. The PPL phosphorylation increases sarcoplasmic reticulum calcium sequestration by enhancing the activity and/or affinity of SERCA-2a for Ca^2+^. This in turn enhances intracellular calcium-cycling capacity and, hence, contractility (Figure 4). 

Additionally, in HFrEF patients, CCM increases phosphokinase G and A related phosphorylation state of TnI, and of myosin-binding protein C in LV and RV, as soon as 30 min after signal delivery and was sustained after 3 months of CCM therapy [24]. Since the sensitivity of the cardiac myofilaments to Ca^2+^ is primarily positively regulated by the phosphorylation state of TnI and of myosin-binding protein, this leads to CCM mediated increased contractility (Figure 4). 

Moreover, the hypo-phosphorylation of titin leads to an increase in stiffness of the myocyte. It has been proven that an increase in both right and left ventricle, with a 21% and 36% rise in total titin phosphorylation (PKA and PKG mediated) observed at 30 min and 3 months post CCM therapy, respectively (positive lusitropy, Figure 4) [7]. 

## 6. CCM Late-Onset Effects: Maladaptive Fetal Gene Remodeling

In pathophysiologic conditions including hypoxia, ischemia, hypertrophy, and atrophy, stressed myocytes return to fetal metabolism which uses carbohydrates as substrates for energy provision in hypoxic environment instead of oxidation of fatty acids. Common features of all of these conditions are extensive protein remodelling, a decrease in the rate of aerobic metabolism in the cardiomyocyte, and a temporary increase in cardiac efficiency. Nonetheless, in failing heart muscle, at a certain point, the fetal gene program is no longer sufficient to support cardiac structure and function [25].

**Figure 5:**
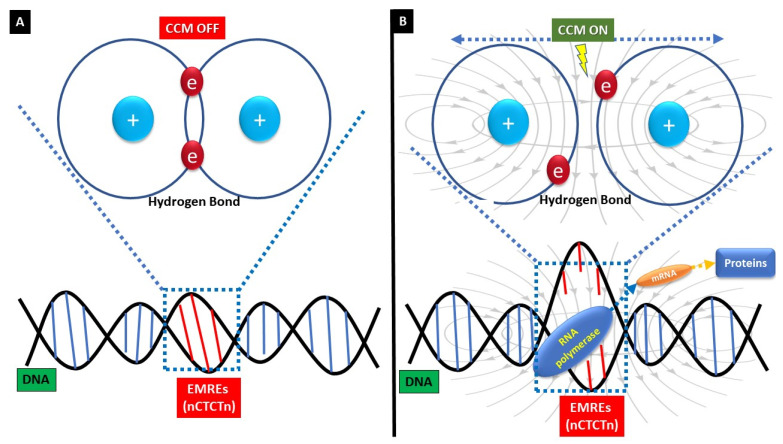
**Schematic representation on CCM action on DNA and protein synthesis.** CCM improves myocardial gene expression by EM field action on specific DNA sequences (nCTCTn, (**A**)). EM fields displace electrons, and this causes transient charging of small groups of DNA base pairs. At the charged sites, disaggregation forces overcome H-bonds. Disaggregation of the two chains at those sites enables transcription (**B**).

CCM improves myocardial gene expression by EM field action on specific DNA sequences (nCTCTn, Figure 5A). EM fields displace electrons, and this causes transient charging of small groups of DNA base pairs. At the charged sites, disaggregation forces overcome H-bonds. Disaggregation of the two chains at those sites enables transcription (Figure 5B). Inserting these EMREs into a promoter of a reporter gene that is unresponsive to EM fields makes that gene EM field-responsive. Removing or mutating these EMREs eliminates the EM field response [10].

CCM reverses the cardiac maladaptive fetal gene program and normalizes expression of key sarcoplasmic reticulum genes (Figure 4). Preclinical studies demonstrated that CCM signal treatment reverses the cardiac maladaptive fetal gene program and normalizes expression of key sarcoplasmic reticulum Ca^2+^ cycling and stretch response genes. Specifically, 3 months on CCM therapy resulted in decreased expression of A- and B-type natriuretic peptides, and p21 Ras, and increased the expression of α-MHC, SERCA-2a, phospholamban, and ryanodine receptors [9]. CCM also attenuated interstitial fibrosis by reducing collagen production and fibroblast differentiation by inhibiting TGF-β1 signaling [26].

**Figure 6:**
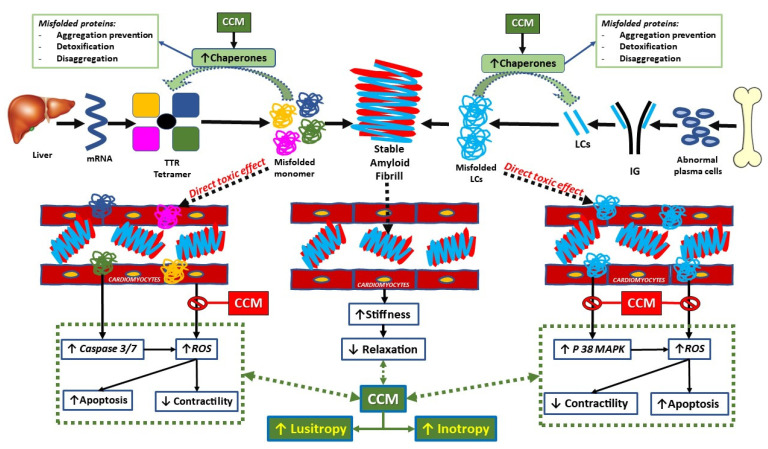
***Cardiac Amyloid phatophysiology and CCM therapeutic effect.*** See explanation in the text. TTR: transtiretin. CCM: cardiac contractility modulation; LC: light chains; IG: immune globulin; ROS: reactive oxygen species; P 38 MAPK: p38 mitogen activated protein kinase.

CCM also acts on several processes which are involved in amyloid cardiomyopathy (Figure 6). CCM-driven normalization of elevated diastolic Ca^2+^ levels in the failing heart might be associated with ROS reductions and activation of CaMKII [8]. CCM decreased the expression of p38 mitogen activated protein kinase (p38MAPK), which is involved in the direct toxic amyloidogenic-mediated oxidative stress, dysfunction, and cell death of cardiomyocytes [27]. 

Remarkably, CCM enhances the transcription of chaperones (such as HSP70), which regulate the balance of protein synthesis and degradation, assist with refolding misfolded proteins, and can protect against cell death in stressful/pathological conditions such as amyloid [28,29]. 

## 7. Cardiac Contractility Modulation Therapy in Amyloid Cardiomyopathy Patients with Heart Failure (AMY-CCM: ClinicalTrials.gov Identifier: NCT05167799)

CCM’s mechanism of action could be beneficial in cardiac amyloidosis but there are no data in this specific clinical setting. To fill this gap in knowledge, we promoted an observational registry whose primary aim is to evaluate the efficacy of CCM in patients with HF and diagnosed TTR amyloidosis. We will focus on TTR, as AL could have different confounding factors, such as more systemic involvement compared to TTR forms, and thus a different prognosis according to specific hematologic treatment.

The Registry has already been approved by competent Ethics Committees and registered on clinicaltrials.gov as the AMY-CCM Registry. The results could bring new evidence on the potential impact of CCM therapy in cardiac amyloidosis as a synergistic therapeutic option.

## Data Availability

No new data were created.
